# Simplistic Attachment and Multispectral Imaging with Semiconductor Nanocrystals

**DOI:** 10.3390/s111110557

**Published:** 2011-11-07

**Authors:** Travis L. Jennings, Robert C. Triulzi, Guoliang Tao, Zachary E. St. Louis, Sara G. Becker-Catania

**Affiliations:** eBioscience, Inc., 10255 Science Center Drive, San Diego, CA 92121, USA; E-Mails: robert.triulzi@ebioscience.com (R.C.T.); guoliang.tao@ebioscience.com (G.T.); soccerrox03@earthlink.net (Z.E.S.L.)

**Keywords:** quantum dot, nanocrystal, IHC, immunohistochemistry, spectral imaging, multiplexing, conjugation, antibody

## Abstract

Advances in spectral deconvolution technologies are rapidly enabling researchers to replace or enhance traditional epifluorescence microscopes with instruments capable of detecting numerous markers simultaneously in a multiplexed fashion. While significantly expediting sample throughput and elucidating sample information, this technology is limited by the spectral width of common fluorescence reporters. Semiconductor nanocrystals (NC’s) are very bright, narrow band fluorescence emitters with great potential for multiplexed fluorescence detection, however the availability of NC’s with facile attachment chemistries to targeting molecules has been a severe limitation to the advancement of NC technology in applications such as immunocytochemistry and immunohistochemistry. Here we report the development of simple, yet novel attachment chemistries for antibodies onto NC’s and demonstrate how spectral deconvolution technology enables the multiplexed detection of 5 distinct NC-antibody conjugates with fluorescence emission wavelengths separated by as little as 20 nm.

## 1. Introduction

Immunohistochemistry (IHC), or the immunological staining and detection of cellular markers in tissue, is a mainstay technique in pathology and diagnostics research and is the current recipient of several new advances in technology; advances which may be capable of evolving IHC from its roots in limited colorimetric staining to a multiplexed fluorescence-based method capable of imaging multiple target antigens within a single tissue section. Quantum dot nanocrystal (NC) fluorescent markers in combination with multispectral imaging (MSI) technology has been a particularly exciting union that has resulted in a respectable number of promising studies over the past few years [1–8].

Quantum dot NC’s are semiconductor particles with dimensions in the 1–10 nm size regime and which exhibit a host of favorable optical properties that make them ideally suited for molecular detection. In particular, NC’s boast narrow, Gaussian-shaped emission spectra notably absent of red-tailing, broad excitation spectra that increase toward the ultraviolet, extinction coefficients and quantum efficiencies which are both impressively high, and a tenacious resistance to photobleaching even under intense excitation [9–13]. When used as a fluorescent probe bound to antigen-derived markers such as IgG antibodies (Ab), NC’s have demonstrated their rather unique utility for multiplexed detection [3,6,7,13–15] and their further potential for antigen quantification [5,8,16,17], proposing a solid alternative to the more traditional but limiting two and three step immunostaining. Thus, NC’s represent a technology capable of reducing sample number and preparation requirements, while simultaneously elucidating more complex cell-cell interactions, protein localization and expression patterns [3]. Despite the many favorable optical properties of NC’s, as a fluorescence-based method in which tissue is the substrate (IHC, *in vivo* imaging, *etc*.) they must yet overcome endogenous autofluorescence associated with tissue sections [2].

The advent of MSI technology has specifically addressed the difficulties surrounding the detection of multiplexed fluorescent markers from the prevalence of background autofluorescence. Multispectral imaging cameras, which may be mounted to a standard camera port on a fluorescent microscope, are capable of replacing individual emission filters optimized to detect one fluorophore each, with a single tunable liquid crystal filter able to detect multiple fluorophores with wavelength resolution down to 3 nm. By creating “spectral libraries” of all involved fluorophores from control samples, the accompanying software is capable of detecting and deconvoluting fluorescence profiles for each fluorophore involved on a *per pixel* basis, thus delivering a high resolution image in which each pixel’s spectrum has been measured and background-subtracted [2]. Therefore, the relationship between NC’s and MSI technologies seems naturally symbiotic, where the antibody-conjugated NC’s provide stable, bright, narrow emission profiles with biological significance, and MSI provides hardware and software capable of detecting and deconvoluting individual signals to reconstruct a meaningful image. The real challenge to achieving high quality images of a tissue sample lies in the particular details of producing the Ab-NC conjugate (conjugation chemistry and format) and the staining protocol (sequential addition or cocktail formulation). Conjugation and staining parameters are capable of dictating either the success or failure of this endeavor.

Although previous reports on the use of NC’s in IHC have been successful in demonstrating the range of 2-plex to 5-plex colorimetric multiplexed staining, achieving these goals required either custom modification of the NC surface [17], unique, sequential staining methods [3,5], or the use of NC conjugates as secondary reagents [6,8,16,18,19], all of which contribute additional complexity and time to the overall process. To successfully implement the mainstream use of fluorescent NC markers into IHC across multiple disciplines such as cancer biology, immunology, and pathology will require a simple, robust procedure for producing Ab-NC conjugates coupled with standard staining techniques which can be applied in a facile yet rapid manner. In this report, we present two simplistic conjugation chemistries that enable expedient NC conjugation to monoclonal antibodies in combination with optimized staining techniques that allow a simultaneous cocktail-style protocol for multiplexed antigen detection.

## 2. Experimental

### 2.1. Materials

All monoclonal antibodies were grown in-house from either tissue culture hybridoma or mouse ascites and purified over protein G affinity column. 100 kDa and 30 kDa molecular weight cutoff concentrators were purchased from Millipore, and desalting spin columns were purchased from Princeton Separations (CS-800). n-hydroxysuccinimidyl ester 6-hydrazinonicotinamide (S-HyNic) and 4-formylbenzide (4FB) were obtained from Solulink, and phospholipids (1,2-distearoyl-sn-glycero-3-phosphoethanolamine-N-[methoxy(polyethylene glycol)-2000] (mPEG)) were purchased from Avanti Polar Lipids. 2-Mercaptoethanol (2-Me), aniline, and sulfobenzaldehyde were purchased from Sigma-Aldrich.

### 2.2. Nanocrystal Synthesis and Modification

The eFluor CdSe/ZnS core/shell NC’s were synthesized using standard high temperature reactions by injection of organometallic precursors into hot coordinating solvents [20–22], with final photoluminescence (PL) emission wavelengths centered near 525, 565, 605, 625, and 650 nm. The nanocrystals were then dispersed into phospholipid micelles using established techniques [23]. Maleimido-activation and 4FB-modification of the lipid layers were performed using proprietary methods and the phospholipid micelle-encapsulated NC’s were then either lyophilized to dryness (maleimide NC’s) or stored in the refrigerator (4FB-NC’s) at 4 °C until use.

### 2.3. Amine-Reactive Chemistry

**Figure 1.**
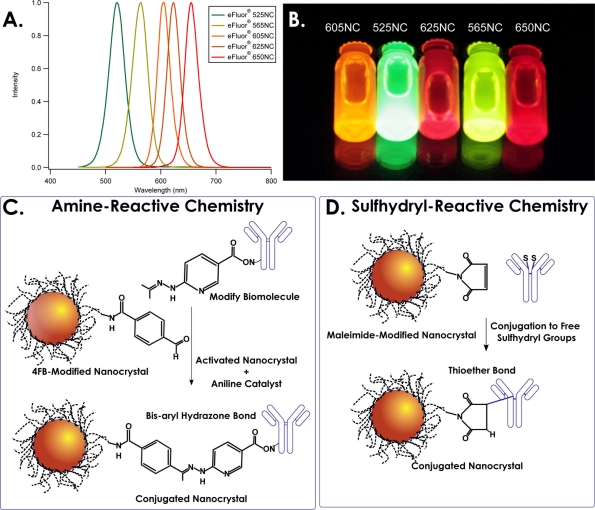
Illustration of conjugation reactions. (**A**) Emission spectra of all 5 NC’s used in this study; (**B**) Photograph of the nanocrystals under ultraviolet excitation in room light. Nanocrystals are composed of CdSe/ZnS and are dispersed into water with a phospholipid micelle coating; (**C**) Schematic of the amine-reactive chemistry in which the lipid-coated nanocrystals have been pre-modified with 4FB for specific catalyzed ligation to a HyNic-modified antibody; (**D**) Schematic of the sulfhydryl-reactive chemistry in which the disulfide linkages near the antibody hinge region have been reduced to free sulfhydryl groups. Conjugation occurs when the sulfhydryl group reacts with an NC surface maleimide to form a thioether bond. NC and Ab dimensions not necessarily drawn to scale.

The amine-reactive chemistry utilizes a bioorthogonal ligation reaction targeting available primary amine groups on the biomolecule and a complementary molecule on pre-activated NC’s. Figure 1(C) describes the process by which the primary amine(s) on the target biomolecule are first modified by incubation with a heterobifunctional NHS-HyNic for 30 min at room temperature. Post-modification, excess HyNic is removed with a de-salting spin column and the HyNic-modified biomolecule is then added to a solution of 4FB-modified NCs. Aniline is added as a catalyst for 4FB-HyNic ligation process [24,25], and the reaction is allowed to proceed for 2 h. The reaction is quenched by the addition of sulfo-benzaldehyde, and the NC-conjugates are then transferred to a 100 kDa centrifuge filter, diluted with 100 mM borate buffer pH 8.4 and buffer exchanged 3 times to remove unreacted antibodies and catalyst. Finally, purified conjugates are microcentrifuged briefly at 1,000 × g for 5 min to remove any undissolved solids and cross-linked materials. As the hydrazine and aldehyde reactants are orthogonal to almost all biological functionalities, they do not alter the subsequent structure or capabilities of other groups present in the NC-bioconjugate.

### 2.4. Sulfhydryl-Reactive Chemistry

The sulfhydryl-reactive chemistry utilizes a maleimide-activated NC surface coating to target reduced thiols in the hinge region of antibodies, as diagrammed in Figure 1(D). The NC’s are stored as a lyophilized powder which includes a proprietary reducing agent. To reconstitute the NC’s, 100 *μ*L of phosphate buffered saline (PBS) pH 7.4 was added, and the mixture warmed in a 60 °C water bath for 30 s or until optically clear. To begin conjugation, 200 *μ*g of monoclonal Ab was pipetted directly into the NC solution and allowed to react for 2 h. At the end of the conjugation reaction, 1 *μ*L beta mercaptoethanol (2-Me) was added and given another 10 minutes to quench any remaining maleimide groups. The NC-conjugates were then transferred to a 100 kDa centrifuge filter, diluted with 100 mM borate buffer pH 8.4 and buffer exchanged 3 times to remove unreacted antibody and 2-Me. The purified conjugates were microcentrifuged briefly at 1,000 × g for 5 min to remove any undissolved materials and cross-linked NC’s. Final NC conjugate concentrations were measured using Beer’s Law and the extinction coefficients given in Table 1.

### 2.5. Multicolor Immunohistochemistry

Male C57Bl mice were euthanized using isoflurane followed by cervical dislocation using IACUC-approved protocols. The spleen was removed and placed in ice-cold 1× PBS, pH 7.2, and rapidly frozen in 2-methyl butane on dry ice for 30 s prior to removing and embedding the spleen in OCT compound. The spleen was stored at −80 °C until sectioning. A 10 *μ*m thick section was cut using a Leica CM3000 cryostat, mounted onto a Superfrost Plus slide, and stored at −80 °C.

Prior to staining, sections were allowed to come to room temperature, fixed for 10 min in 100% acetone, and then gently washed with 1× TBS, pH 7.4. Nonspecific binding sites within the tissue were blocked with 1% BSA in TBS for 1 h at room temperature. Experiments were performed to optimize the concentration of NC-labeled antibodies on sections of spleen after which a cocktail of NC-labeled antibodies was prepared by diluting each of the NC-labeled antibodies to its predetermined optimal concentration. The five NC-labeled antibodies were diluted to the following concentration and combined in 1% BSA in TBS as follows: eF525-B220/20nM, eF565-PECAM-1/20nM, eF605-CD11b/5nM, eF625-CD4/5nM, and eF650-CD11c/1nM. Both positive and negative control slides were prepared using each of the NC-labeled antibodies alone as well as one slide with blocking solution and no antibodies to serve as an autofluorescence control. The blocking solution was removed from the section, and the five-color antibody solution (or single colors) was overlaid and covered lightly with Parafilm and incubated in the dark at 4 °C overnight in a humidified chamber. Sections were washed with three changes of 1× PBS and coverslipped with Fluoromount G mounting medium. The edges of the coverglass were sealed with clear nail polish.

Fluorescence intensity was imaged using an Axiovert 200 microscope equipped with a Nuance EX spectral imager (CRI). All images were taken using a 20× Plan Apochromat objective and using a 460 nm excitation, 475 nm dichroic, and 500 nm long pass emission filter. Images were taken from 500 to 750 nm at 10 nm intervals using an exposure time of 50–100 ms. A spectral library was constructed from the individually stained slides consisting of the representative spectrum of each of the NC’s and the autofluorescence signal. By assigning a NC to each representative spectrum and subtracting out the autofluorescence spectrum, the Nuance software was able to differentiate each individual NC signal. The five-color slide was then imaged and each NC spectra identified and assigned a pseudocolor in the combined image.

## 3. Results and Discussion

### 3.1. Chemistry

The proper assembly of a nanoscale material, such as a NC, with a biomolecule for detection purposes would be optimal with the assembly method that produces the conjugate with the highest and most desirable activity. “Activity”, in this case, may be defined as fastest reaction rate, highest avidity, brightest signal, or lowest non-specific binding, to name a few. Therefore, mounting interest is being placed on the development of new conjugation chemistries or techniques that may give the researcher control over the conjugate’s activity by controlling properties such as NC:molecule stoichiometry, site-selective ligation, or molecular orientation [26] with an emphasis on bioorthogonal ligation chemistries [27] or chemistries that target rare events within the biomolecule for higher selectivity.

The amine-reactive chemistry described here (Figure 1(C)) is an example of a successful bioorthogonal chemistry applied to NC-Ab conjugate formation. In this conjugation, the antibody is first modified with a heterobifunctional crosslinking molecule (NHS-HyNic) that targets the prevalent amine functionalities found throughout proteins, and presents the bioorthogonal hydrazine moiety outward for ligation to the 4FB aldehyde on the NC. This reaction is catalyzed by the presence of aniline with rates between 10^1^ and 10^3^ M^−1^ s^−1^ in mildly acidic to neutral conditions [24,25], which has yielded NC conjugates to monoclonal Ab’s in our laboratories in as short a time as 30 minutes that performed equivalently to reactions given 2 h. The hydrazine and aldehyde moieties are orthogonal to essentially all native functionalities found in proteins, and as such will react cleanly only with each other to leave the rest of the biomolecule relatively undisturbed.

The sulfhydryl-reactive chemistry described in Figure 1(D) describes the exceedingly simple NC-maleimide reaction to an Ab reduced at the hinge region. Because the presence of sulfhydryl containing moieties is somewhat rare, their use as a handle in Ab conjugations provides a well-controlled point of attachment that yields site-selective assembly of these NC-Ab conjugates. The NC’s have been pre-activated with maleimide functionalities at the extremity of the PEG phospholipid micelle and then stored with a proprietary co-reducing agent as a lyophilized powder under vacuum. Although the functionalization of NC’s with a maleimide group for sulfhydryl-ligation has been accomplished before through the use of heterobifunctional crosslinkers such as succinimidyl-4-(N maleimidomethyl)cyclohexane-1-carboxylate (SMCC) [29,30], these NC’s were modified immediately prior to use, and any unutilized NC would have to be discarded due to hydrolysis of the maleimide group. The maleimide-NC’s described here have been stabilized for at least one year in the lyophilized format and are available for immediate use with no activation/purification steps prior. Also, due to the presence of a stable reducing agent lyophilized with the NC’s, the conjugation reaction is greatly simplified to the basic steps of (1) reconstituting the NC and reducing agent into buffer; (2) adding the biomolecule (must contain a cysteine, disulfide, or equivalent), and finally (3) purification.

We found that likely due to the increased surface area available for conjugation, the larger NC’s (eF650NC) required a higher Ab:NC ratio of 4, and the smaller NC’s (eF525NC) showed sufficient conjugate activity at a ratio of only 2. This trend was maintained for both chemistries. It should also be noted that, while higher Ab ratios may yield better performance with increased avidity, free Ab may not be sufficiently purified without resorting to the use of more rigorous purification methods like size-exclusion chromatography, that significantly increases the time and instrumentation requirements to produce a conjugate.

### 3.2. Multiplexed Staining

A comparison of the many different methods used in the literature for the multiplexed staining of tissue belies the complexity but importance relegated to this portion of the experiment. The highest successes for multiplexed staining (as determined by the degree of “plexing”) have been reported by Liu *et al.* [6] with 4-plex staining, and both our group and Fountaine *et al.* [3,7] who accomplished 5-plex staining.

Fountaine *et al.* accomplished 5-plex staining of formalin-fixed paraffin embedded (FFPE) tonsil tissue by designing a sequential staining method whereby five different streptavidin (SAV)-coated NC’s were used to do the multiplexing. Their method employed a three-step staining procedure for each NC in which a primary Ab was incubated first, followed by washing then incubation with a biotinylated secondary Ab, followed by washing and staining with the particular SAV-NC, followed by an avidin-biotin blocking step before repeating the entire procedure for the next NC. Interestingly, this research group also reports a three-fold loss in signal intensity when the NC is bound directly to the secondary antibody in an attempt to eliminate the SAV-Biotin complexing step, which justified the need for three incubation steps per NC. The authors do not discuss disassociation kinetics involved with the multi-layer sandwich assay, but it has been shown that SAV-NC conjugates possess a ten-fold increase in “off” rate kinetics compared to the native SAV-biotin interaction [31], which would potentially deteriorate signal and increase the non-specific background staining because all specificity in this assay is based upon SAV-Biotin. Liu *et al.*, on the other hand, were successful in eliminating SAV-Biotin from the sequential method and streamlining the staining process further.

Liu *et al.* accomplished 4-plex staining using NC’s conjugated to secondary Ab’s in a semi-sequential manner to increase throughput of the staining protocol [6]. By first incubating the sample with primary Ab’s of two different isotypes, they were able to perform a 4-plex NC staining of FFPE tissue with only two sequential rounds of NC-Ab staining where each round consisted of: primary Ab incubation (2 isotypes), washing steps, NC-Ab secondary isotype specific staining (2 NC colors stained simultaneously), then blocking before the second round of NC-Ab staining. Two aspects in particular set the Liu staining approach apart from earlier efforts: they successfully utilize NC’s conjugated to secondary antibodies (this attempt produced sub-optimal results for Fountaine *et al.*), and they negate concern over disassociation kinetics by cross-linking NC-Ab-antigen complex using common tissue fixatives. Thus, the efforts of Liu *et al.* represent a significant step forward in allowing multiplexed staining of NC’s on tissue. Yet, this begs the question: why not assemble the monoclonal Ab directly onto the NC and skip all subsequent staining steps?

Presumably, the primary reason for research groups to go the route of secondary staining or rely on SAV-biotin interactions is the commercial availability of NC’s bound to secondary Ab’s or SAV-NC conjugates. Xing *et al.* also speculate that direct conjugation to monoclonal Ab’s may deactivate the Ab binding properties, be negatively impacted by serum or other carrier proteins, and increase costs because each NC-Ab conjugate may be used against only one marker [17]. If, however, reactive NC’s were available that made custom NC conjugates to monoclonal Ab’s a feasible option in terms of time, difficulty and price, *and* the custom conjugates were capable of producing similar results to either the secondary Ab or SAV-NC conjugates, then the ability to perform multiplexed staining by cocktail formulation in a single step should be a compelling reason to move toward this approach. By offering two conjugation chemistries to either the non-targeted but ubiquitous amine functionalities or the site-specific and rare sulfhydryl groups, we allow for difficulties associated with potential binding properties to be overcome by the researcher. Also, since the chemistry is easily performed on relatively small amounts of Ab (200 *μ*g Ab, and could likely be scaled down), the cost per experiment is not unreasonable. Arguments related to interference caused by other proteins should affect secondary Ab conjugates equally, and in our observations, the inclusion of BSA as a blocking protein in NC conjugates has not negatively affected our results. When BSA is included in the cocktail we actually observe improved specificity and resolution, albeit we have noticed that brightness is affected slightly which is overcome by a small increase in NC concentration.

To effectively stain all 5 proteins in mouse spleen, matching the appropriate NC with Ab is the first and perhaps the most critical design consideration in a multiplexed experiment. As shown in Table 1, the extinction coefficients vary greatly from small-to-large NC’s, where the smallest NC’s have the lowest extinction values, which means that small NC’s like 525NC will absorb and therefore emit far less light than their larger 650NC counterpart over the same integration time. When matching each NC with an Ab, the Ab affinity and expected antigen expression levels were taken into account such that the most prevalent target was matched with the 525NC, and the least prevalent target matched with the largest 650NC. Further, as Table 1 describes, optimal staining concentrations follow a clear trend of decreased concentration with the larger NC’s, which is a direct ramification of the extinction coefficient logic combined with antigen expression levels to achieve similar fluorescence intensity of all targets. Once NC-Ab conjugates were designed and the optimal concentration levels determined, a cocktail solution was mixed and used to stain tissue samples for simple multiplexed detection and imaging.

### 3.3. Multispectral Imaging

After the challenge of NC-Ab design and direct conjugate incubation has been achieved, detecting and distinguishing each fluorophore marker becomes the next hurdle due to the possibility of spectral overlap and autofluorescence complications. Although the use of NC’s for multiplexing has been shown to substantially alleviate the difficulties associated with resolving different fluorescent markers in relatively clean polymer systems [32], trying to accurately decipher between more than 3 colors in mammalian tissue amidst a broad spread of background luminescence is difficult and requires a more advanced optical system and deconvolution software than is bundled with most fluorescence microscopes [33].

A full description of the deconvolution algorithms designed and bundled with multispectral imaging software is beyond the scope of this paper, but is essentially the mathematical disentangling of different known spectral profiles weighted by their contribution to the total spectrum, *i.e.*, If multiple wavelengths of light have combined to produce a unique intensity spectrum, those same wavelengths may also be deconstructed from the total spectrum back to their component parts. This “spectral unmixing” process is made possible mathematically because emitted light combines linearly to produce a spectrum containing the sum of all wavelengths and their relative intensities [2]. The caveat is that an understanding of the individual fluorescence spectra involved must be obtained *a priori* to performing the multiplexed experiment through the creation of a spectral library. A spectral library is created by imaging each fluorophore individually (including unstained tissue to gauge autofluorescence) which will later allow the software to recognize the signature spectrum of each fluorophore during the unmixing process. Because tissue autofluorescence may be considered its own spectral profile, assuming homogeneity across the tissue sample, then autofluorescence subtraction is as easy as recognizing and subtracting its weighted contribution across the image.

**Figure 2.**
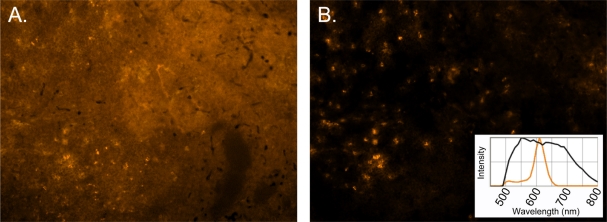
Autofluorescence subtraction for 605NC-CD11b stained mouse spleen. (**A**) shows an image of tissue prior to background subtraction when looking through a microscope objective. Although NC fluorescence may at times be visible on individual macrophage cells, fully-resolved CD11b+ cells are not distinguishable until after autofluorescence spectral unmixing and subtraction have taken place; (**B**) is the resulting fluorescence image after the autofluorescent background has been eliminated, leaving behind only fluorescence signal derived from positively-stained 605NC-CD11b macrophage cells in the tissue. Inset: Example of autofluorescence emission profile (black) and 605NC emission profile (orange) derived from the tissue sample and used to create the spectral library.

Figure 2(A) shows a simple single-color stain of mouse spleen using a 605NC-CD11b conjugate as the image would appear through the objective using typical excitation and emission filters specific to the 605NC. Although a single NC could likely be imaged cleanly by adjusting exposure time, this image shows the prevalence of autofluorescence and the difficulty discerning positively-stained cells. Figure 2(B) shows the same image after applying the appropriate library (figure inset) to spectrally unmix the 605NC and autofluorescence signals. Clearly, multispectral imaging combined with unmixing algorithms are powerful tools for detecting and deciphering specific fluorescence signals out of the autofluorescent milieu. We refer the interested reader to several articles on the use of spectral imaging and spectral unmixing [1,2,34,35].

**Figure 3.**
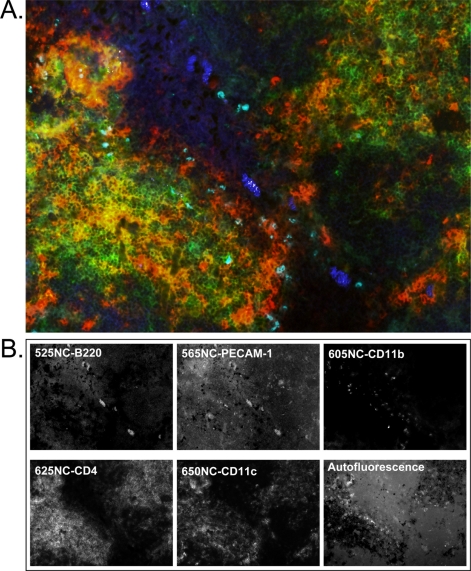
(**A**) Five-color multiplexed image (psuedo-colored composite) of mouse spleen after staining with NC monoclonal Ab direct conjugates, imaging with the CRI Nuance multispectral imager, and spectral unmixing to remove autofluorescence; (**B**) Individual NC fluorescence signals resulting from spectral unmixing. Each of the individual images is assigned a pseudo-color (autofluorescence is assigned black) and overlaid to form the composite image above. The conjugate/color scheme is as follows: 525NC-B220/blue, 565NC-PECAM-1/yellow, 605NC-CD11b/aqua, 625NC-CD4/green, 650NC-CD11c/red.

As a final test of the abilities of the NC-Ab direct conjugates to stain their individual targets from a cocktail formulation and utilizing the multispectral imager to effectively unmix all fluorescence wavelengths, we performed a 5-plex staining of mouse spleen tissue. Figure 3(A) shows a representative composite image of 5-color multiplexed staining after spectral unmixing and autofluorescence removal. In this image, the structure/function relationship of multiple immune cell types is shown with the use of 5-color staining. The 525NC-B220 conjugate (blue) highlights a B-cell center, which is closely associated with leukocyte and endothelial cells stained by 565NC-PECAM-1 (yellow). PECAM-1 (CD31) is a surface protein expressed on all leukocyte and endothelial cells in low levels, and functions in cell-cell adhesion and signal transduction. The 605NC-CD11b conjugate (aqua) targets macrophage cells which express integrin *α_M_*, and the 625NC-CD4 conjugate (green) targets the CD4 molecule which is expressed by the majority of thymocytes found in the mouse spleen. The 650NC-CD11c conjugate (red) is specific for integrin protein *α_X_* found on activated T cells and dendritic cells, and appears as individual cells either surrounding the B cells or throughout the tissue. Because very little co-localization of multiple NC wavelengths appears in the final image, this suggests that each NC-Ab conjugate was specific for each particular surface marker. Although in this experiment the usage of a particular chemistry to produce each conjugate was random, the matching of each NC with an Ab for conjugation was decided based on expected expression levels and NC extinction coefficients as discussed previously. Designing the conjugates in this manner allowed us to achieve similar brightness levels for each marker.

Figure 3(B) shows the unmixed spectra specific to each emission profile (including autofluorescence) which have been recombined to form the composite 5-color image found in part A. In the single-color images, each marker is delineated from all of the other NC wavelengths present, further demonstrating the utility of multispectral imaging when multiple fluorescent markers are involved. Indeed, no co-localization was expected because each NC-Ab conjugate was directed at a specific cell type. However, the clear ability to image each NC conjugate separately suggests that studies requiring co-localization of markers may work equally well, paving the way for the elucidation of cell-cell and even protein-protein interactions in future studies.

## 4. Conclusions

The studies described here detail two novel conjugation chemistries allowing for the rapid and facile attachment of nanocrystals to biological molecules using either ubiquitous amine groups found on most biomolecules for general attachment strategies, or less common sulfhydryl moieties for more targeted attachment. Both chemistries are used to conjugate five separate NC’s to 200 *μ*g each of five different monoclonal antibodies using simple conjugation and purification protocols. The conjugates were tested individually to stain mouse spleen tissue and a spectral library was created for spectral unmixing by the multispectral imager. A cocktail formulation was mixed which contained each of the NC conjugates at its optimized concentration, and the cocktail was used for direct staining of mouse spleen tissue. An epifluorescence microscope with multispectral imager attachment was used to view and photograph the slides, and spectral unmixing by the supplied software showed that the system was capable of not only resolving each NC-Ab conjugate from neighboring emission spectra, but also simultaneously removing background autofluorescence to produce a meaningful image of cellular patterns where each cell was identified by cell type and location. The combination of narrow emission spectra and unique conjugation chemistry provided by the NC’s, with multispectral imaging, spectral unmixing and background autofluorescence subtraction demonstrates the clear potential that these technologies may bring to pathology and diagnostics research.

## Figures and Tables

**Table 1.** Selected optical properties and details of Ab-NC conjugates used in this study. Quantum efficiency measurements were measured relative to either FITC in absolute ethanol (eF525NC) or rhodamine 6G in absolute ethanol. Extinction coefficients listed are relative to the wavelength for the first exciton absorption feature [28].

**NC**	**λ***_P L_*	**QY**	**Extinction Coefficient (M**^−1^**cm**^−1^)	**Antibody**	**Conjugation Chemistry**	**Staining Concentration**
525NC	527	63%	5.57 × 10^4^	B220	Amine-Reactive	20 nM
565NC	566	60%	1.12 × 10^5^	PECAM-1	Amine-Reactive	20 nM
605NC	608	64%	2.85 × 10^5^	CD11b	Sulfhydryl-Reactive	5 nM
625NC	628	70%	5.21 × 10^5^	CD4	Sulfhydryl-Reactive	5 nM
650NC	651	68%	1.12 × 10^6^	CD11c	Sulfhydryl-Reactive	1 nM
